# Investigation the potential use of silver nanoparticles synthesized by propolis extract as N-acyl-homoserine lactone-mediated quorum sensing systems inhibitor

**DOI:** 10.3906/sag-2004-148

**Published:** 2020-06-23

**Authors:** Sedef İLK, Gamze TAN, Ezgi EMÜL, Necdet SAĞLAM

**Affiliations:** 1 Department of Immunology, Faculty of Medicine, Niğde Ömer Halisdemir University, Niğde Turkey; 2 Department of Biology, Faculty of Science and Letters, Aksaray University, Aksaray Turkey; 3 Department of Nanotechnology and Nanomedicine, Institute of Graduate School of Science and Engineering,Hacettepe University, Ankara Turkey

**Keywords:** silver nanoparticles, quorum sensing, virulence factor, antimicrobial agent, propolis

## Abstract

**Background/aim:**

Quorum sensing (QS) is a chemical communication process that bacteria use to regulate virulence. Inhibition of QS (antiQS) overcomes the pathogenicity of bacteria. Silver nanoparticles (AgNPs) have been used as antimicrobials against pathogens, but have not been used against QS-mediated bacterial infection. Also, studies have been carried out on the inhibitory effects of propolis based structures on pathogen growth, but no studies have been found on their potential use as QS inhibitor. The present study aims to investigate the synthesis and characterization of silver nanoparticles (AgNPs) reduced with propolis extract (P–AgNPs) and evaluation of their antimicrobial and, for the first time, antiQS activity.

**Materials and methods:**

P–AgNPs were synthesized using with different volumes (1, 2.5 and 5 mL) of propolis extract (PE) by biological method via reduction of silver nitrate. Synthesized P–AgNPs were characterized in terms of hydrodynamic, chemical, morphological, physical, and antioxidant properties. Disc diffusion and flask incubation assays were used to evaluate the antimicrobial effect against Gram–negative bacteria (*Escherichia coli*, *Proteus mirabilis,* *Proteus vulgaris,* *Salmonella typhimurium, Enterobacter aerogenes, Pseudomonas aeruginosa)* and Gram–positive bacteria (*Staphylococcus aureus, Streptococcus mutans, Bacillus thuringiensis*) and QS–regulated biofilm activity against biosensor strain *Chromobacterium violaceum* CV026.

**Results:**

AgNPs were successfully synthesized by biological method via PE. The violacein pigment production based on the QS system was greatly inhibited by the P–AgNPs (inhibition zones: 16.22-21.48 mm and violacein inhibition: 63.16 ± 2.4-75.24 ± 3.5 %) without interfering with the growth of bacteria, which is the first report on the antiQS effect of P–AgNPs.

**Conclusion:**

Our results suggest that P–AgNPs may be potentially used to inhibit bacterial physiological processes due to the signal molecules regulates important collective behavior of bacteria. The development of such nontoxic biomaterials may have great potential to evaluate for the new medicinal substance that inhibits the pathogenic biofilms.

## 1. Introduction

Nanotechnology is the science of achieving the materials more functional by taking advantage of their nanodimensional properties. Due to this dimension, they can be used in many different ways with more sufficient and unique functions. Surface properties are the most favored and attractive parts of current nanoscience at the nanoscale. In addition, improving the materials with many different modification methods makes the nanostructure more biocompatible and using them with natural and biological components are one of the advantageous and practical ways for biomedical and biological applications. 

Metal nanoparticles such as silver, gold, titanium, iron, etc. are one of the most used materials among all the nanostructures. They have many different specific usage areas due to their unique natures. The medical science is seeking new promising approaches with the rising importance of bacterial resistance against most of the antibiotics [1]. Especially, AgNPs have always aroused great interest among the researchers with their antimicrobial, antifungal, and antiviral properties. Nanosilver systems have gained a reputation due to their respectable activity against many microorganisms, even in case of a low dose [2]. Synthesis and usage are very inexpensive and practical. Silver shows very little toxicity for humans with a small number of doses [3]. 

Propolis is a sticky substance collected by honeybees. Propolis has been used traditionally as a natural remedy for the treatment of a variety of diseases through the inclusion of a large number of active compounds, such as flavonoids. Through contained flavonoids, propolis becomes highly effective against antibiotic resistant bacterial strains [4,5]. Propolis is commercially available in several forms in folk medicine due to its medical advantages such as antiinflammatory, antioxidant, antimicrobial, and cytostatic effects. The antimicrobial activity of propolis against many pathogens has been studied and widely known for many years [6]. Recently, the inhibitory effects of propolis based nanostructures on pathogens have been studied [7,8], but still, there is a lack of a nanotechnological knowledge about propolis and its medicinal and biological activities with nanostructures. 

Interfering with the bacterial communication system (Quorum Sensing) is one of the alternatives to bacterial biofilm formation inhibition [9, 10]. QS is described as a signaling mechanism in bacteria that is an important regulator for biofilm formation as well as other bacterial behaviors [11]. The antiQS system provides a new strategy to prevent diseases of bacteria regulated by signal molecules at the early stages of bacterial infections [12]. An important advantage of the antiQS applications for the development of bacterial resistance is that they do not impose a high selective pressure as antibiotics. Both AgNPs and propolis show great antimicrobial activities, but the main difference between these microbial effects is the way they are used.

While propolis can demonstrate its antimicrobial effects by its flavonoids, AgNPs demonstrate them with the reducing agents which are used in the synthesis of them [13]. In literature, there have been several antimicrobial studies of propolis and AgNPs and also their combined form by bacterial growth dependent manner, however there has been not any studies of P–AgNPs formulation on the effect of bacterial signal molecule inhibition which regulates biofilm formation of several pathogenic bacteria. For this purpose, in this study, we aimed to develop the potential use of silver nanoparticles synthesized by propolis extract (PE) as bacterial signal molecule inhibitor (antiQS) molecule. Thus, in this work, we synthesized biocompatible, nontoxic, and nonreactive nanoparticulate material based on AgNPs covered with propolis which inhibits bacterial growth against several human pathogens and, as the first time, blocks bacterial signal molecules against biomonitor strain *Chromobacterium violaceum CV026*. P–AgNPs with different volumes (1, 2.5 and 5 mL) of PE were prepared by biological synthesis method. The study also includes the characterization of the materials in terms of physicochemical structure-property-functionality relationships. This work opens the way to controlling bacterial behavior by affecting their communication using nontoxic and inexpensive substances included nanomaterials.

## 2. Materials and methods

### 2.1. Materials

Silver nitrate (AgNO3) was purchased from Fluka. Sodium borohydride (NaBH4), Luria Bertoni (LB) agar, Müller-Hinton medium were purchased from Sigma-Aldrich. Other chemicals and solvents were of analytical reagent grade. Propolis was obtained from the Black Sea region of Turkey by Professor Necdet Sağlam.

### 2.2. Synthesis of P–AgNPs

For the synthesis of P–AgNPs, firstly, PE was prepared as follows: propolis pieces (30g) were grounded well in pestle. Propolis was dissolved and extracted by ethanol (70%) and then, this solution was sonicated (Bransonic 220) using ultrasonic bath and stirred at room temperature for 1 h. Then the propolis solution was collected and stored at +4 °C. Following that, the solution was mixed overnight in the dark then filtered through Whatman filter paper. In this study, we tested the effect of PE volume in constant amount of AgNPs formulation by means of physical, chemical and morphological structure and also biological activity as antimicrobial and antiQS molecule. Thus, we evaluated the different volumes of PE (1, 2.5, and 5 mL) for the synthesis of P–AgNPs. NaBH4 was used for the chemical reduction of silver salt into AgNPs. The synthesis steps can be described as follows: 30 mL of 2 mM NaBH4 was prepared in ice bath on a stir plate. Then, 2 mL of 1 mM AgNO3 was rapidly added into the solution to reduce silver ion and to obtain a yellow solution of AgNPs. For the synthesis of the P–AgNPs, a certain volume of the PE (1, 2.5, and 5 mL) was added to the AgNO3 (10-3M) aqueous solution. The solution was stirred for 15 min at room temperature. The AgNPs prepared using different amounts of PE (1, 2.5, and 5 mL) were called P–AgNPs1, P–AgNPs2, and P–AgNPs3 in our manuscript, respectively.

### 2.3. Characterization of hydrodynamic diameters and concentration

The zeta potential, size, and polydispersity index (PDI) of the prepared P–AgNPs1, P–AgNPs2, and P–AgNPs3 were analyzed in a capillary cell by dynamic light scattering (Zetasizer Nano ZS, Malvern Instruments, UK) at 25 °C. The measurements were done in triplicate. The results were demonstrated as values with a mean ± standard deviation. The concentrations of prepared P–AgNPs1, P–AgNPs2, and P–AgNPs3 were determined by inductively coupled plasmon resonance (ICP-MS) (Perkin Elmer, Optima 4300 DV). The experiments were replicated 3 times. 

### 2.4. Trolox equivalent antioxidant capacity (TEAC)

For the TEAC assay, firstly ABTS (2,2-Azino-bis-3-ethylbenzothiazoline-6-sulfonic acid) was dissolved in a buffer of acetate and added into potassium persulfate according to Ozgen, Reese [14] with some modifications. The prepared ABTS containing potassium per sulfate solution was diluted in 20 mM sodium acetate buffer (pH 4.5) to an absorbance of 0.700 ± 0.01 at 734 nm. Finally, 2.97 mL of the ABTS∙+ solution and 30 mL of pure PE and prepared nanoparticle solutions (P–AgNPs1, P–AgNPs2, and P–AgNPs3) were mixed and allowed to stay for 10 min, and the absorbance was determined at 734 nm by using UV/Vis spectrophotometer (PG, T60). The measurements were done in triplicate.

### 2.5. Characterization of hydrogen bonding

The hydrogen bonding of pure PE, AgNPs, P–AgNPs1, P–AgNPs2, and P–AgNPs3 were evaluated by using Fourier Transform Infrared Spectroscopy (FTIR, Perkin Elmer Nicolet 520 spectrophotometer, Boston, USA). The samples were analyzed in the range of 450-4000 cm-1 with 4 cm-1 resolution, using 16 scans for the FTIR analysis. The measurements were replicated 3 times. 

### 2.6. Morphological observations

Scanning Electron Microscopy (SEM) (Zeiss, Evo 40) was used for the evaluation of the surface morphology of the synthesized P–AgNPs1, P–AgNPs2, and P–AgNPs3. For the SEM measurements, nanoparticles were mounted onto aluminum pin type stubs (diameter: 12 mm) with carbon tape, and then nanoparticles were coated with palladium–gold. Transmission Electron Microscopy (TEM) (FEI Tecnai G2 Spirit BioTwin, USA) was used for the characterization of the inner morphology of the P–AgNPs1, P–AgNPs2, and P–AgNPs3. The nanoparticle solutions were sonicated for 10 s, and 10 mL of each 1 was taken and then placed onto the copper grid and dried at room temperature for the TEM analysis. Then, it was analyzed by the High Contrast Transmission Electron Microscope (CTEM).

### 2.7. Antimicrobial activity of P–AgNPs

The following microorganisms including *Escherichia coli* (*E. coli*) ATCC 25922, *Staphylococcus aureus* (*S. aureus*) ATCC 25923, *Streptococcus mutans* (*S. mutans*) ATCC 25175, *Proteus mirabilis* (*P. mirabilis*) ATCC 14153, *Salmonella enterica serotype typhimurium* (*S. typhimurium*) SL 1344, *Proteus vulgaris *(*P. vulgaris*) ATCC13315, *Bacillus thuringiensis *(*B. thuringiensis*), *Enterobacter aerogenes *(*E. aerogenes*) *ATCC13048*, and* Pseudomonas aeruginosa *(*P. aeruginosa*) ATCC 27853 were used as the tested microorganisms. Bacteria were subcultured on LB agar at 37 °C for 24 h. Antimicrobial activities of PE, AgNPs and the synthesized P–AgNPs1, P–AgNPs2, and P–AgNPs3 against the bacteria were evaluated by the test of the disc diffusion according to the reference method of the National Committee for Clinical Laboratory Standards (NCCLS) [15]. The microorganisms’ turbidity was modified by 0.5 McFarland standards. The bacteria culture (100 mL) was swabbed (106 cells/mL) onto Müller-Hinton agar of Petri plate and filter discs (6 mm in diameter) within nanoparticles were placed on the inoculated agar and incubated at 37 °C. The disc with gentamicin (10 mg/disc) as positive control and only solvent (diluted ethanol) as negative control were used. All tests were replicated 3 times. The results were demonstrated as the mean diameter of the inhibition zone in mm ± standard deviation (mean ± SD).

### 2.8. AntiQS bioassay of nanoparticles

The *Chromobacterium violaceum* (*C. violaceum*) CV026 as reporter strain and *C. violaceum* ATCC 12472 as biomonitor strain were used to determine the antiQS properties of P–AgNPs1, P–AgNPs2, and P–AgNPs3, only PE and AgNPs. The suspension of bacteria was subcultured by overnight culture (30 °C) in Luria-Bertani (LB) broth. Disc diffusion test was carried out to evaluate the antiQS activity of synthesized nanoparticles. For the test, firstly, C6-HSL (0.25 mg/mL) as signal molecules was added to LB agar. The strain *C. violaceum* CV026 was swabbed onto the prepared Petri plates. Then, discs were taken onto the Petri plates and the samples (20 mL) were loaded and incubated at 30 °C for 30 h. Gentamicin as positive control and only solvent as negative control (diluted ethanol) were used. Halo formation with a purple background around the discs revealed that the tested samples exhibited an antiQS effect. All tests were replicated 3 times. The results were demonstrated as the mean diameter of the inhibition zone in mm ± standard deviation (mean ± SD).

The quantitative evaluation of violacein inhibition of the prepared samples was evaluated *C. violaceum* ATCC 12472. The violacein concentration inhibition was determined according to the reference literature proposed by İlk, Sağlam [16]. The samples’ suspension was poured to the *C. violaceum* ATCC12472 culture. As negative and positive control, only solvent and gentamicin (10 mg/mL) were used, respectively. After incubation, the violacein extraction was done. Culture from each sample was centrifuged (13552 RCF, 10 min) in order to precipitate the insoluble violacein and bacterial cells. Then, the phase of supernatant was discarded, and the pellet was centrifuged. The supernatant’s absorbance was measured at 585 nm using a UV/Vis spectrophotometer (PG, T60). The test was done 3 times. The results were demonstrated as the mean diameter of the inhibition zone in mm ± standard deviation (mean ± SD). The violacein inhibition (VI) was expressed as a percentage. The percent inhibition of violacein was evaluated by the following formula:

VI% = ((control(OD585)–test(OD585))/control(OD585)) × 100 (Eq. 1)

### 2.9. Statistical analysis 

Statistical analysis to compare size and zeta values of P–AgNPs and antimicrobial inhibition zone values (mm) against 2 groups (Gram–positive and Gram–negative) bacteria of P–AgNPs was performed using “IBM SPSS Statistics ANOVA” and “IBM SPSS Statistics-independent sample t test” programs, respectively. The significance level was accepted as P <0.05.

## 3. Results

### 3.1. Characterization of P–AgNPs

**Figure 1:**
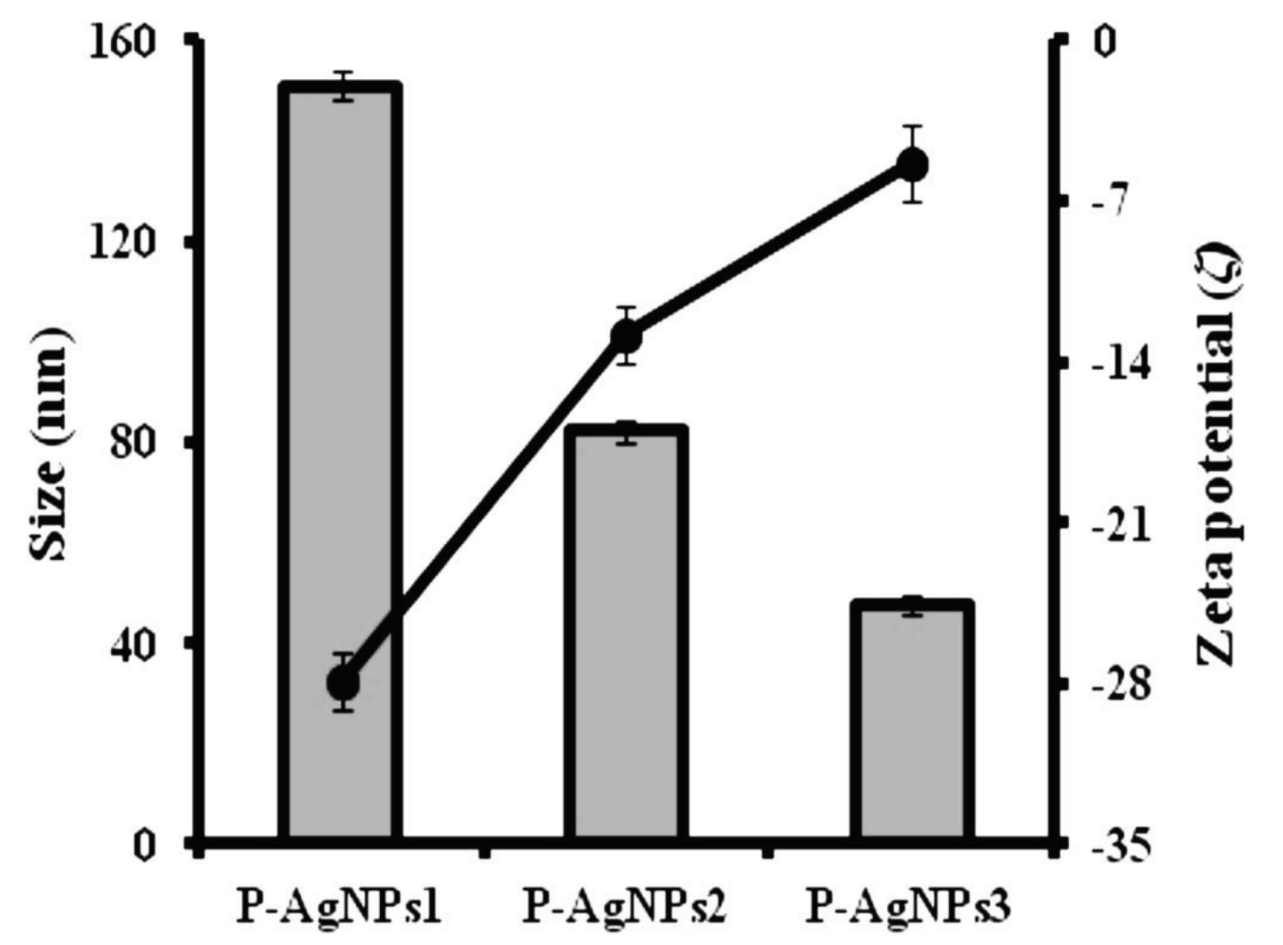
Average size (grey bars) and surface charges (black squares) of P–AgNPs1, P–AgNPs2, and P–AgNPs3 in different volume of PE (1, 2.5, and 5 mL, respectively) (P > 0.05).

P–AgNPs were prepared using bioactive components of PE that reduced AgNO3. The results indicated that when the amount of AgNO3 was constant, the hydrodynamic diameter of nanoparticles was highly influenced by the PE volume (Figure 1). A linear relationship was observed: increasing PE volumes in AgNPs formulations led to decreasing values of particle size and polydispersity index. The formulations containing 1, 2.5, and 5 mL of PE in a constant amount of AgNO3 suspension (1 × 10-3 M) presented low polydispersity index (PDI ≤ 0.4, data not shown) within average sizes of 150.90, 82.26, and 47.72 nm for the P–AgNPs1, P–AgNPs2, and P–AgNPs3, respectively. The aggregation behavior of P–AgNPs was observed with values higher than 2.5 mL amount of PE (PDI: 0.82) in a constant amount of AgNPs. When zeta potential value studies of P–AgNPs were examined, nanoparticles indicated to have negative zeta-potential values from -5.37 to -27.9 mV at different volumes of PE (1 to 5 mL), illustrating that P–AgNPs had negative surface energy as suggested due to excess negative charges of AgNO3 molecules after bonding with propolis hydrogen ions (Figure 1). When the results of size values of P–AgNPs were compared with their zeta potential, the values of each AgNPs were not statistically significant (P = 0.0982). The negative charge values of nanoparticle decreased linearly with the increasing volume and mass ratio of PE in AgNPs formulations. Nanoparticles (P–AgNPs1, P–AgNPs2, and P–AgNPs3) were prepared by using increasing volumes of PE (1, 2.5, and 5 mL) at a constant amount of AgNO3. However, after nanoparticle fabrication, the content of Ag molecules was changed (Table 1) due to the repulsion between the groups of positive charges of propolis and negative charge of AgNO3. 

**Table 1:** Concentration of Ag molecules (mg/mL) in P–AgNPs.

Nanoparticles	mg/mL
P–AgNPs1	121 ± 2
P–AgNPs2	114 ± 1
P–AgNPs3	99 ± 5

**Figure 2:**
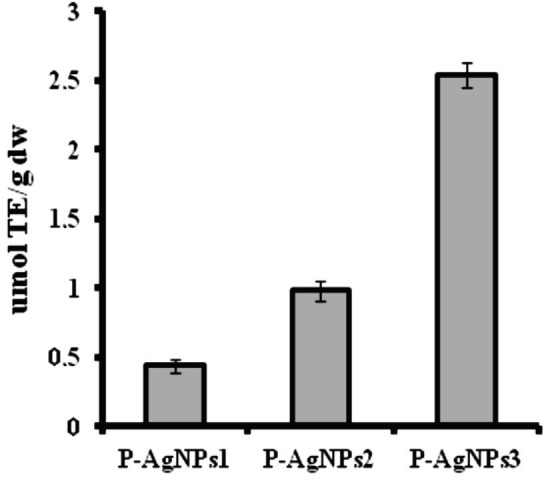
Trolox equivalent antioxidant capacityof P–AgNPs1, P–AgNPs2, and P–AgNPs3 in different volume of PE (1, 2.5, and 5 mL, respectively).

The TEAC assay was used to evaluate the antioxidant capacity of P–AgNPs1, P–AgNPs2, and P–AgNPs3 in different PE volumes to donate protons. The TEAC assay indicated that the phenolic group’s capacity of PE donated and saturated hydrogen in order to stabilize free radicals (Figure 2). P–AgNPs1 (0.44 mmol TE/g dw), P–AgNPs2 (0.98 mmol TE/g dw), and P–AgNPs3 (2.54 mmol TE/g dw) were capable of reducing higher values of TEAC molecules [14].

**Figure 3:**
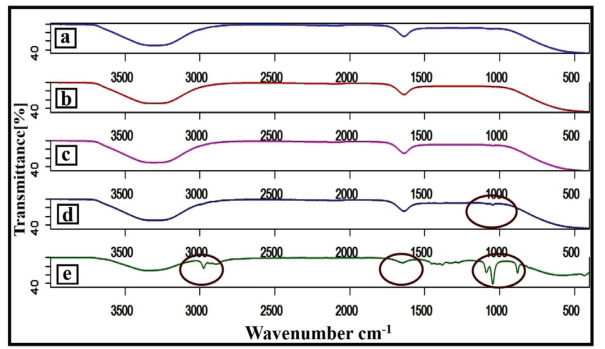
FTIR spectrum of P–AgNPs1 (a), P–AgNPs2 (b), P–AgNPs3 (c), AgNPs (d), and PE (e).

FTIR analysis was performed to investigate possible reactions between pure PE, AgNPs, and P–AgNPs1, P–AgNPs2, and P–AgNPs3 at different volumes of PE (Figure 3). The FTIR spectrums of P–AgNPs1, P–AgNPs2, and P–AgNPs3 (Figures 3a, 3b, and 3c) displayed similar absorbance peaks: 3250 cm-1 (O-H stretch) and 1650 cm-1 (C=O bands). The AgNPs spectrum (Figure 3d) indicated typical 3250 cm -1 (O-H stretch), 1654 cm-1 (C=O bands), 1601 cm-1 (C=C), and 1054 cm-1 (C-O stretch). The PE spectrum (Figure 3e) shows the characteristic absorption bands and specific molecular peaks of its chemical structure: 3300 cm-1 (O-H stretch), 2970 cm-1 (C-H stretch), 1675 cm-1 (C=O bands), 1604 cm-1 (C=C), 1157 cm-1 (C-O-C), 1067 cm-1 (C-O stretch). Furthermore, P–AgNPs1, P–AgNPs2, and P–AgNPs3 spectrum displayed that the C=O absorption band at 1675 cm-1 of propolis shifted to 1654 cm-1 and the C-O-C and C-O stretch of propolis completely disappeared at 1157 cm-1 and 1067 cm-1, respectively (Figures 3a, 3b, 3c, and 3e) [17]. 

**Figure 4:**
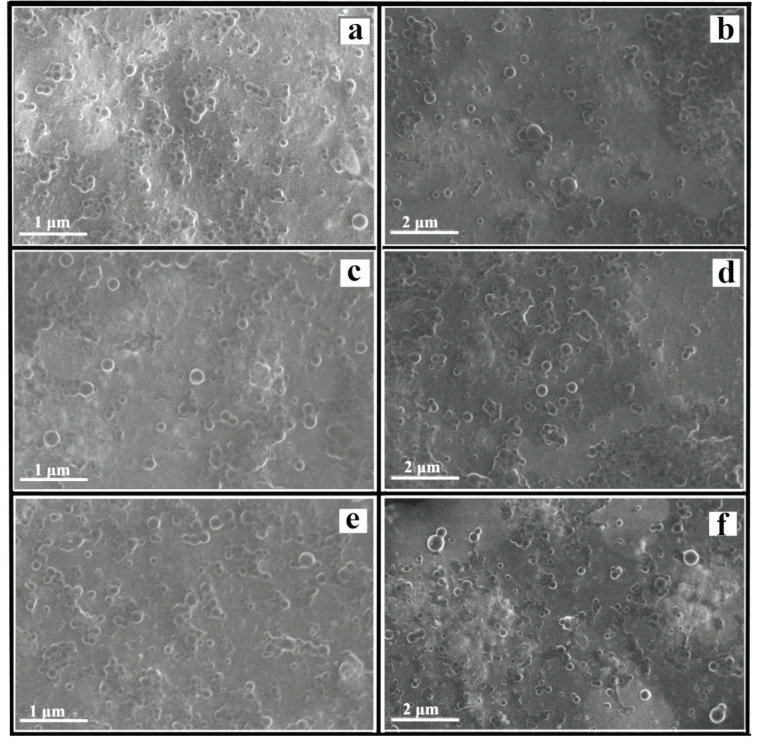
Surface morphology of P–AgNPs1 (a and b), P–AgNPs2 (c and d), and P–AgNPs3 (e and f ).

**Figure 5:**
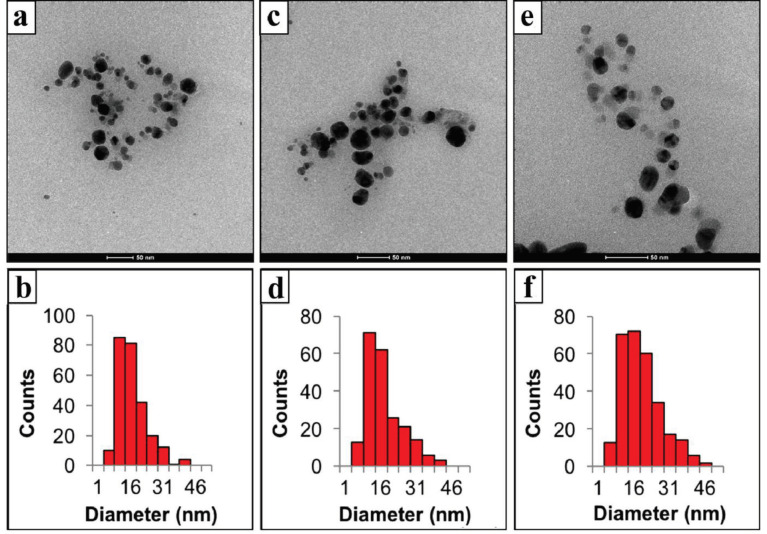
TEM images and size distribution of P–AgNPs1 (a and b), P–AgNPs2 (c and d), P–AgNPs3 (e and f ).

From the SEM images of P–AgNPs, it was observed that P–AgNPs1, P–AgNPs2, and P–AgNPs3 are spherical in shape, uniform, and polydisperse (Figure 4). SEM images indicated that an insight into the average size of particles, which is about 145.90 ± 6.1 nm, 80.62 ± 4.5 nm and 41.27 ± 5.4 nm for the P–AgNPs1 (Figures 4a and 4b), P–AgNPs2, (Figure 4c and 4d) and P–AgNPs3 (Figure 4e and 4f), respectively. The results indicated that the high volume of PE leads to a decrease in the P–AgNPs1, P–AgNPs2, and P–AgNPs3 size. Similar results were also obtained in zeta size measurements (Figure 1). On the other hand, in our study, the inner morphology of the developed P–AgNPs has been investigated using TEM (Figure 5). Analysis of the TEM images indicated that the mean particle size was approximately P–AgNPs1: 14.16 ± 6.74 nm (n = 255) (Figure 5a and 5b), P–AgNPs2: 14.65 ± 7.46 nm (n = 216) (Figures 5c and 5d), and P–AgNPs3: 16.42 ± 8.19 nm (n = 288) (Figures 5e and 5f), respectively. AgNPs were dense and spherical, with a size range between 14–16 nm (Figure 5) [18]. The TEM micrograph also demonstrates that the AgNPs were surrounded by a thin layer of PE. 

### 3.2. Bioactivity assays of synthesized nanoparticles

Antibacterial activity was screened around AgNPs, pure PE and synthesized P–AgNPs1, P–AgNPs2, P–AgNPs3 inoculated discs against bacteria and expressed as a diameter of inhibition zone (mm). The data present that there is a significant difference in sensitivity between the Gram–positive and Gram–negative bacteria in the tested samples (Table 2). The antibacterial properties of samples are higher against Gram–negative (Gram-) bacteria than against Gram–positive bacteria (Gram+). In Gram- bacteria, *P. aeruginosa *was found to be the most sensitive bacteria in the tested microorganisms against fabricated P–AgNPs1, P–AgNPs2, and P–AgNPs3 (14.98 ± 0.95, 20.37 ± 1.12, 27.96 ± 1.44 mm, respectively). Moreover, *B. thuringiensis *was found to be the most resistant bacteria on all the tested P–AgNPs1, P–AgNPs2, and P–AgNPs3 (10.89 ± 0.88, 13.17 ± 0.91, 16.85 ± 0.97 mm, respectively) in Gram+ bacteria. *S. aureus *and *S. mutans* demonstrated nearly similar sensitivity against all tested compounds. 

**Table 2:** Table 2. Antimicrobial activities of P–AgNPs, PE, AgNPs expressed as inhibition zone diameter (mm) (P <0.05). Statistical analysis (IBM SPSS Statistics-independent sample t test) of antimicrobial inhibition zones (mm) P–AgNPs1, P–AgNPs2, P–AgNPs3 in 2 groups of Gram–positive and Gram–negative microorganisms.

Microorganisms	P–AgNPs1	P–AgNPs2	P–AgNPs3	PE	AgNPs	Control	Gentamicin
Escherichia coli (Gram-)	13.27 ± 0.92	18.72 ± 1.14	24.65 ± 1.22	11.02 ± 0.87	12.51 ± 0.91	-	30.56 ± 0.24
Salmonella typhimurium (Gram-)	12.86 ± 0.86	17.35 ± 1.02	22.79 ± 1.16	10.86 ± 0.82	11.24 ± 0.90	-	33.12 ± 0.28
Proteus mirabilis (Gram-)	10.95 ± 0.72	14.36 ± 0.98	18.29 ± 1.07	10.07 ± 0.80	10.16 ± 0.86	-	31.47 ± 0.22
Proteus vulgaris (Gram-)	11.88 ± 0.81	15.42 ± 1.06	19.08 ± 1.09	10.92 ± 0.78	11.04 ± 0.88	-	32.45 ± 0.23
Pseudomonas aeruginosa (Gram-)	14.98 ± 0.95	20.37 ± 1.12	27.96 ± 1.44	12.16 ± 0.85	13.21 ± 0.89	-	27.19 ± 0.20
Enterobacter aerogenes ­(Gram-)	12.65 ± 0.82	17.18 ± 0.99	22.44 ± 1.21	12.23 ± 0.89	12.18 ± 0.91	-	30.15 ± 0.21
Staphylococcus aureus (Gram+)	11.82 ± 0.84	14.09 ± 0.90	17.75 ± 1.04	10.52 ± 0.82	10.61 ± 0.84	-	29.27 ± 0.20
Streptococcus mutans (Gram+)	11.05 ± 0.89	13.94 ± 0.88	17.06 ± 0.95	10.59 ± 0.82	10.83 ± 0.82	-	30.05 ± 0.21
Bacillus thuringiensis (Gram+)	10.89 ± 0.88	13.17 ± 0.91	16.85 ± 0.97	10.02 ± 0.84	10.43 ± 0.84	-	29.72 ± 0.21

**Figure 6:**
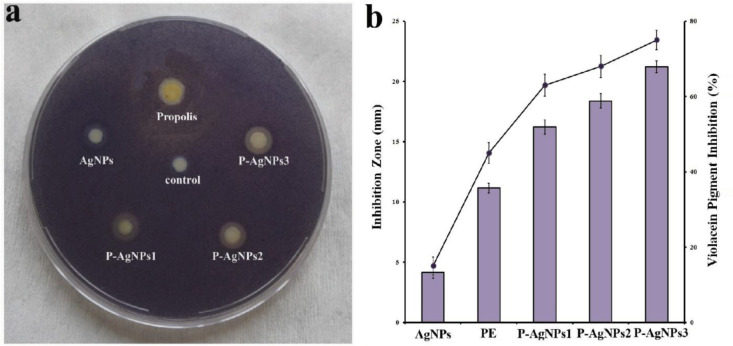
Disc diffusion assay for the antiquorum sensing activity (a) inhibition zone (mm) (light purple bars), and quantitative determination of violacein inhibition (%) (black squares) (b) of AgNPs, PE, P–AgNPs1, P–AgNPs2, and P–AgNPs3.

The antiQS activity of pure AgNPs, PE, and P–AgNPs with different PE contents was determined against *C. violaceum CV026*. In the disc diffusion test, all the nanoparticles showed antiQS activity against *C. violaceum CV026* (Figure 6a). Three concentrations of tested P–AgNPs displayed the inhibition zone of violacein production within the range of 16.22–21.48 mm (Figure 6b). P–AgNPs3 containing the highest PE content presented a significant pigment reduction compared to other contents of PE according to radios of clearance zone. The highest values of inhibition of pigment production were obtained from P–AgNPs3*.* In addition, interestingly the pigment inhibition of pure PE displayed lower antiQS activity than all the P–AgNPs and pure AgNPs illustrated the lowest antiQS effect than PE (Figures 6a and 6b). 

To determine quantitative signal molecule reduction, the changing of purple pigment concentration was also evaluated against *C. violaceum *ATCC 1247. All the tested P–AgNPs showed a significant inhibition for the violacein concentration within the range of 63.16 ± 2.4-75.24 ±3.5% (Figure 6b). Similarly, pure PE showed lower inhibition of violacein production (45.14 ± 3.2%) when compared to P–AgNPs. With regards to results, P–AgNPs3 exerted a higher pigment inhibition (75.24 ± 3.5%) against *C. violaecum CV026 *and antimicrobial activity (Table 2) against tested pathogen bacteria when compared to P–AgNPs2 and P–AgNPs1, which demonstrated antibacterial activity along with the antiQS activity.

## 4. Discussion

In this study, P–AgNPs were synthesized using green synthesis method, which is known as a rapid, cheap, and easy fabrication route. It is easy to make changes in parameters in this method and therefore, surface charge and size values of P–AgNPs can be controlled by changing parameters such as PE amount. The hydrodynamic parameters are presumed to be the essential properties for the nanoparticle evaluation because they affect the capacity of stability, encapsulation, and release of nanoparticles. Additionally, the zeta potential and particle size induce antimicrobial properties against microorganism [19]. Nanoparticles within decreasing size present increasing surface area/volume ratio and encapsulation capacity, which leads to high release efficiency. However, nanoparticles which have decreasing nanosized diameter can also present more aggregate structure for long-term applications. The nanoparticles with low polydispersity index (PDI ≤ 0.1-0.6) properties can solve this aggregation problem and achieve maximum stability. All concentrations of tested P–AgNPs had PDI value up to 0.4 within sizes ranged from 47.72-150.90 nm. However, the PE volume above 2.5 mL presented aggregate structure (PDI ≥ 0.8) due to blocking behavior of repulsive forces with the AgNPs due to decreasing of inter-space between nanoparticles, thus leading to aggregation. Similar results were observed by Raffa, Iannuzzo [20] for AgNPs with a high loading of agents. The size of P–AgNPs can be manipulated easily for industrial applications. It could be caused by the fact that in nanoparticle engineering systems, propolis was in equilibrium with the constant amount of AgNO3, and above this volume, PE reached saturation within AgNO3 molecules. Hence, P–AgNPs with increasing PE amounts were presented that the increasing values surface charge. Zeta potential provides key information for the antimicrobial effect of agents through the interaction with microbial cell surface [21]. P–AgNPs1–3 showed that negative zeta-potential values from -5.37 to -27.9 mV. Similar results were also reported: AgNPs have negative surface charges at pH 4.5 with zeta potential values between –5 and -27 mV [22–24].

It was reported that the reducing power is generally related to the presence of a reductant [25]. Propolis is considered to have high antioxidant activity. However, several environmental conditions, especially light and heat during the preparation process can influence its antioxidant capacity and limit its application. Biological active compounds like propolis in nanoparticle formation can prevent their antioxidant nature due to hydrogen bonding between them and nanoparticle core or shell material. In our study, we found that P–AgNPs had higher antioxidant capacity (0.44 mmol TE/g dw and 2.54 mmol TE/g dw) with increasing PE volumes from 1 to 5 mL, respectively. From the results, it is observed that the propolis with different volumes can keep its antioxidant capacity following nanoparticle synthesis. The hydrogen bonding interaction between them was also evaluated by FTIR measurements. The spectroscopy results indicated that the intramolecular hydrogen bonding was structured between PE and AgNPs after the formation of P–AgNPs [18]. Surface properties of nanomaterials have an important effect on drug release kinetics [26]. Interestingly, when the results of the size and surface morphology of P–AgNPs compared with the results data of TEM micrograph, the size of P–AgNPs within increased amount of PE decreased from 150.90–47.72 nm, but the size of AgNPs increased from 14.16–16.42 nm. We suggest that it could be caused by the fact that propolis with the constant amount of AgNO3 was in equilibrium. 

The antibacterial activities of P–AgNPs against Gram- bacteria showed higher zone size than Gram+ bacteria (P < 0.05). It may be assumed that Gram- bacteria have a simple cell wall structure which has many pores that can allow disturbing cell wall and the cytoplasm with different molecules. We suggest that the results of antimicrobial activity of P–AgNPs1–3 may be associated with the disruption of low peptidoglycan structure of bacteria cell wall since lipid–lipid interactions provide liquidity of Gram- bacteria cell wall and membrane. This could stem from the differences in the cell wall structure of Gram–negative and Gram–positive, considering all of the incubations were carried out in the same conditions. Gram- and Gram+ bacteria contain peptidoglycans as part of their cell walls, but peptidoglycan layers are thin and low in Gram- and thick and quite compact in Gram+ [27–29]. However, *P. mirabilis *displayed stronger resistance than other bacteria on the tested pure PE, AgNPs, P–AgNPs1, P–AgNPs2, and P–AgNPs3 because *P. mirabilis *can elongate itself and secrete a polysaccharide when in contact with surfaces. This mechanism of *P. mirabilis* provides resistance to items such as antimicrobial agents [30]. Furthermore, in Gram+ bacteria, *B. thuringiensis *showed the highest zone against all the P–AgNPs1–3. This result indicated that the *Bacillus* species might be a form of endospores that are more resistant to biocidal agents than other bacteria strains [31]. From the results, it was observed that P–AgNPs1, P–AgNPs2, and P–AgNPs3 within an increasing amount of PE resulted in a significant increase in the antimicrobial zone of all the tested microorganisms. P–AgNPs3 (within 5 mL PE) showed maximum zone of inhibition against all tested bacteria than P–AgNPs1 and P–AgNPs2 (containing 2.5- and 1-mL PE, respectively). This mechanism was presumed that compounds of the flavonoids of PE ensure to lysis the cells of bacteria by binding to the components of the cell wall and causing disruption of cytoplasm [32]. Also, natural antioxidant PE within AgNO3 can show a strong antimicrobial effect against both Gram- and Gram+ bacteria.

The results obtained from antiQS studies exhibited the potential use of AgNPs covered with PE, for the first time, as antiQS agents owing to inhibiting the violacein production without inhibition of bacterial growth. The obtained antiQS properties of the P–AgNPs may contribute to the bioactive capacity of polyphenolic rich compounds of PE. The nontoxic and nonreactive antimicrobial and antiQS potential of the novel biobased nanoparticle engineering systems could be useful further for the development of the therapeutics on the pathogenesis of the tested bacteria. 

In conclusion, for the first time, significant inhibition against the quorum sensing regulated pigment production of the bacteria *C. violaceum CV026* was observed using the novel P–AgNPs without the addition of any chemical active agents. Although the exact mechanism for the observed antiQS effect of the P–AgNPs is still to be unraveled, this may be related to the affinity of hydroxyl groups such as phenolic contents including flavonoids, phenolic acids, anthocyanins, and several aromatic acids of propolis on the AgNPs surface. Since the autoinducer for the pigment production of *C. violaceum CV026* also regulates many other important physiological behaviors of bacteria, it is expected that these new nanoparticles may be potentially used to inhibit those physiological processes, including biofilm formation. Besides the applications as packaging materials, medical textile, or various biomedical devices, also such sustainable nanoparticles may have the potential in the development of new antivirulence drugs or additive.

## Conflict of Interest

The authors declare that they have no conflict of interest.

This study was presented at the Taiwan-Turkey Science Summit entitled “Translation of Cells, Nanomaterials and Signaling Molecules into Regenerative Medicine” between April 1 to 3, 2018.
